# Efficacy of an Internet-based intervention with self-applied exposure therapy in virtual reality for people with panic disorder: study protocol for a randomized controlled trial

**DOI:** 10.1186/s13063-023-07536-1

**Published:** 2023-08-12

**Authors:** Josephine Schultz, Anna Baumeister, Stella Schmotz, Steffen Moritz, Lena Jelinek

**Affiliations:** Department of Psychiatry and Psychotherapy, University Medical Center Hamburg-Eppendorf, Martinistrasse 52, 20246 Hamburg, Germany

**Keywords:** Online intervention, CBT, Anxiety, Panic disorder, Internet, Guided self-help, Exposure

## Abstract

**Background:**

Due to several treatment barriers, many individuals with panic disorder do not receive evidence-based treatment. One promising option to narrow this treatment gap is Internet-based psychotherapy, which has been shown particularly effective in guided formats. Still, there remains room for improvement to make these digital therapies more accessible, cost-efficient, and aligned with best practices for in-person interventions (e.g., exposure). The smartphone app “Invirto – Treatment for Anxiety” offers digitally guided, evidence-based treatment of panic disorders including virtual reality (VR) for exposure therapy. The aim present study is to investigate the efficacy, safety, and acceptance of Invirto in comparison to a care-as-usual (CAU) control group.

**Methods:**

We plan to conduct a randomized controlled trial with two conditions (intervention vs. CAU), three assessment times via online surveys (t0: baseline; t1: 3 months after baseline; t2: follow-up assessment 6 months after baseline), and a total of 128 participants with a clinical diagnosis of panic disorder (symptoms must be experienced ≥ 1 year). Recruitment will take place via email, phone, and the study website. The primary outcome will be the change in anxiety symptoms as measured by Beck’s Anxiety Inventory from t0 to t1. Secondary outcomes will be the change in anxiety symptoms (measured by the Panic and Agoraphobia Scale, PAS; Questionnaire on panic-related Anxieties, Cognitions and Avoidance, ACA), depressive symptoms (measured by the Beck-Depression-Inventory, BDI-II), treatment satisfaction (measured by the Client Satisfaction Questionnaire, CSQ-8; Treatment Adherence Perception Questionnaire, TAPQ-adapt; Positive and Negative Effects of Psychotherapy Scale, PANEPS-I), psychological flexibility (measured by the Acceptance and Action Questionnaire-II, AAQ-II), and dissociation during VR exposure (measured by an adapted version of the Peritraumatic Dissociative Experiences Questionnaire, PDEQ-adapt). Participants in the intervention group will receive access to the intervention (Invirto) right after t0, while the CAU group will receive access to Invirto after t1. We expect a larger change in both the primary and secondary outcomes from t0 to t1 in the intervention group in comparison to the CAU group.

**Discussion:**

This study is one of the first to evaluate an Internet-based intervention for people with panic disorder that includes self-application of VR exposure therapy. The findings are expected to extend the body of knowledge about effective Internet-based treatment options for people with panic disorder. The empirical and clinical implications and the limitations of the study are discussed.

**Trial registration:**

DRKS00027585 (www.drks.de/drks_web/), date of registration: 13 January 2022.

**Supplementary Information:**

The online version contains supplementary material available at 10.1186/s13063-023-07536-1.

## Background

### Panic disorder and treatment strategies

People with panic disorder suffer from recurrent and unpredictable anxiety attacks that are accompanied by severe physical symptoms such as feelings of suffocation, rapid heartbeat, or chest pain. They often make significant changes in their behavior to avoid future anxiety attacks and experience severe limitations in their daily life activities [1]. The 12-month prevalence for panic disorder is 2–3% in the general population [2, 3]. Panic disorder is associated with high indirect and direct health care costs due to patients’ assumption that a physical illness is causing their alarming symptoms [4], resulting in frequent, inconclusive, and costly physical examinations and emergency medical admissions. Due to the direct and indirect costs to the health care system, anxiety disorders (all ICD-10 F4x- diagnoses) generated the fourth highest aggregate costs across the EU—74 billion Euros in 2010 [5].

According to German [6] and international guidelines such as the National Institute for Clinical Excellence (NICE; [7]), cognitive behavioral therapy (CBT) that includes exposure therapy is the first-line approach in the treatment of panic disorders (with or without agoraphobia). With and/or without the support of a therapist, the patients expose themselves to anxiety-provoking situations and gain new insights into their alarming physical symptoms and their need for safety strategies and avoidance [8]. Whereas Foa and Kozak [9] emphasize the mechanism of habituation in their “emotional processing theory,” Craske [10] highlights the importance of the approach of inhibitory learning and inhibitory regulation to maximize the effectiveness of exposure therapy.

### Internet-based psychotherapy

Although psychotherapy has been proved effective with large effect sizes compared to no treatment [11], many people with panic disorder never receive psychological treatment [12]. Reasons for this treatment gap may include barriers to treatment, which include a shortage of trained therapists, high treatment costs, and the shame and stigma around seeking psychological help [13]. One option to address these barriers is to offer psychotherapy in the form of Internet-based cognitive behavioral therapy (iCBT). Internet-based therapy enables treatment flexibility independent of time and place and also provides a cost-effective way to counteract treatment barriers [14]. iCBT offers low-threshold access to evidence-based and guideline-compliant treatment for various mental disorders. Most commonly, iCBT includes structured guided (accompanied by a therapist) or unguided (self-help interventions) digital treatment programs. In the case of structured interventions, the content is presented either on an Internet platform or through a smartphone app.

In recent years, numerous studies have evaluated Internet-based therapies for various mental disorders, including depression [15], obsessive–compulsive disorder [16], and anxiety disorders [17]. In reviewing the effectiveness of iCBT, cross-disorder studies have demonstrated a positive impact of Internet-based treatment programs with a medium effect size [18]. The amount of therapeutic contact influences the effectiveness of iCBT; unguided self-help interventions (no contact between patient and therapist except for baseline assessment) showed the smallest effect size compared to minimally guided interventions (minimal contact beyond the assessment to assist with use of the self-help intervention) and mostly guided interventions (regular contact between the patient and therapist; [19]).

Regarding anxiety disorders, meta-analytic research shows that psychological interventions delivered via smartphone devices can significantly reduce anxiety symptomatology with moderate to large effect sizes [20]. Firth et al. [21] found significantly greater reduction in anxiety symptoms with smartphone interventions than control conditions (*g* = 0.33). However, effect sizes from smartphone interventions were significantly larger when compared to waitlist/inactive controls (*g* = 0.45) than to active control conditions (*g* = 0.19).

Meta-analyses of guided iCBT programs for panic disorder have found large mean between-group effects on panic severity when compared to waitlist or control conditions [22–24]. In a meta-analysis in which iCBT was directly compared to face-to-face CBT, Carlbring et al. [25] found equivalent efficacy between the two modalities regarding panic symptoms, indicating that the two treatment formats are equally effective.

Exposure and interoceptive exposure are two of the most effective components of face-to-face CBT [26, 27]. Exposure treatment can be effectively represented digitally. Kim et al. [28] investigated the efficacy of a self-training in virtual reality (VR) for patients with social anxiety disorder (SAD) and showed that the VR program provided significant improvements in anxiety symptoms and positive changes in social components such as total speech length, subjective nervousness, and subjective confidence.

### Invirto therapy

The smartphone app “Invirto – Treatment for Anxiety” (“Invirto” for short; developed by Sympatient Ltd.) allows digitally guided, evidence-based treatment of panic disorders. It aims to enable patients to tackle their anxiety symptomology themselves, reduce functional limitations caused by the panic disorder, and improve their quality of life. Invirto includes more than 12 h of digital therapeutic content divided into 8 modules with audio and video content. See Table 1 for more information about the content of each module of Invirto. One component of the treatment is digital exposure therapy in VR. Invirto offers virtual reality exercises that mirror the situations that people with panic disorder find challenging and often avoid. These include simulated situations such as taking a subway or bus ride, standing in a long checkout line at the supermarket, being underground in a tunnel, or using an elevator. It also includes interoceptive exposure exercises to re-evaluate the individual’s physical symptoms of anxiety. Each VR exposure exercise is guided by an audio-recorded therapist and can be performed for up to 90 min. Although several iCBT applications for anxiety disorders have been developed in recent years, to date Invirto is the only digital therapy app that uses VR.

**Table 1:** Modules and content of Invirto

Module #	Content and aim of module
1	Provide overview of treatment, explain psychotherapy
	Explain use of the app
	Emulate a therapeutic relationship
	Explain panic disorder, reducing stigma
	Manage expectations
2	Visualize limitations in everyday life due to anxiety (create cognitive dissonance about the status quo)
	Define problems, symptoms, and fears
	Validate the difficulty of change
	Discuss costs and benefits of changing behavior
	Define goals for the intervention
3	Healthy and pathological anxiety
	Self-reinforcing feedback loops
	Identification of conditions of developing and maintaining factors of anxiety
	Self-observation of anxiety situations and occurrence of anxiety
4	Problems due to avoidance/safety behavior
	Breathing technique for panic attacks, relaxation techniques
	Autonomic nervous system
	Sympathetic and parasympathetic nervous systems
5	Typical thoughts in anxiety
	Effects of anxiety thoughts
	Exploration of one’s own anxiety thoughts
6	Rationale for exposure
	Exercise in virtual reality
7	Understanding the function of emotions
	Understanding the relationship between emotion avoidance and fear
	Understanding diversity of emotions
	Understanding emotions as indicators of needs
	Learning to accept emotions
8	Review of the treatment and what has been achieved
	Advice on how to deal with risky situations
	Preparation of an emergency kit
	Tips for further practice
	Planning for the next phase of exercises

The main objective of the planned study is to investigate the efficacy of Invirto in comparison to a care-as-usual (CAU) control group as well as its safety and acceptance over the intervention period of 12 weeks. The primary outcome is the change in the Beck Anxiety Inventory (BAI) from baseline (t0) to post (t1) assessment after 3 months. Secondary outcomes to investigate efficacy include anxiety symptoms (measured by the Panic and Agoraphobia Scale, PAS), quality of life (measured by the Quality of Life–global item, WHOQOL-BREF), depressive symptoms (measured by the Beck Depression Inventory, BDI-II), and psychological flexibility (measured by the Acceptance and Action Questionnaire-II, AAQ-II), as well as body-related anxiety, catastrophizing cognitions, and mobility avoidance (measured by the Questionnaire on panic-related Anxieties, Cognitions and Avoidance, ACA). Acceptance will be measured by the Client Satisfaction Questionnaire (CSQ-8) and the Treatment Adherence Perception Questionnaire (TAPQ-adapt). Safety will be assessed by the Positive and Negative Effects of Psychotherapy Scale for Internet-based Interventions (PANEPS-I) as well as the Peritraumatic Dissociative Experiences Questionnaire (PDEQ-adapt) to assess dissociation during VR exposure. The CAU group will receive Invirto after assessment at t1. We hypothesize that anxiety symptoms will decrease more in the Invirto group from t0 to t1 compared to the CAU group and that Invirto will be a safe and accepted intervention. In addition, we expect the decrease in anxiety symptoms in the intervention group to be sustained from t1 to t2. Furthermore, we expect a decrease in both the primary and secondary outcomes from t1 to t2 in the CAU group.

## Methods

### Study design

**Fig. 1:**
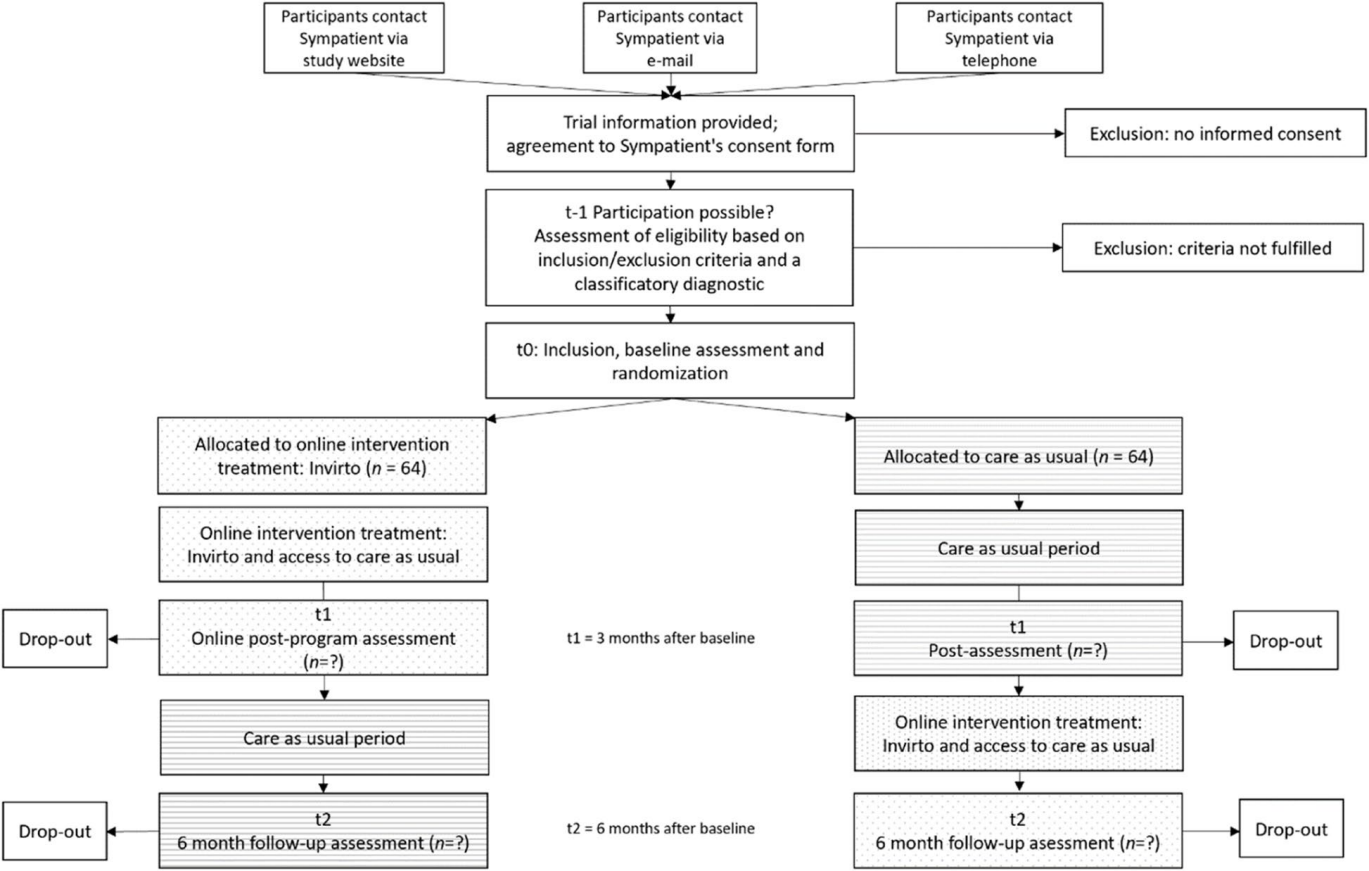
Study flow

The study will be a randomized controlled trial (RCT) investigating the efficacy of guided Internet-based treatment for patients with panic disorder in comparison to a CAU group. As shown in Fig. 1, the study design is a parallel-design randomized waitlist-controlled clinical trial. Participants in the intervention group will receive access to the intervention (i.e., Invirto) right after t0, while the CAU group will receive access to Invirto after the first intervention period of 3 months (t1). Participants will be assessed online via the online survey platform Qualtrics® at baseline (t0), post intervention (t1, 3 months after baseline), and follow-up (t2, 6 months after baseline). See Table 2 for a schedule of enrollment, interventions, and assessments.

**Table 2:** Schedule of enrollment, intervention, and assessments

	**Study period**			
**Time point**	**t-1 Enrollment**	**t0 baseline assessment**	**t1 post assessment**	**t2 follow-up assessment**
***Eligibility screen***	X			
***Informed consent***		X		
***Randomization***		X		
**Access to intervention**				
Invirto (intervention group)		X		
Care as usual (control group)			X	
**Primary outcome measures**				
Beck Anxiety Inventory (BAI)		X	X	X
**Secondary outcome measures**				
Panic and Agoraphobia Scale (PAS)		X	X	X
Beck Depression Inventory-II (BDI-II)		X	X	X
Questionnaire on panic-related Anxieties, Cognitions and Avoidance (ACA)		X	X	X
The Acceptance and Action Questionnaire-II (AAQ-II)		X	X	X
Quality of Life–global item (WHOQOL-BREF)		X	X	X
Questionnaire to evaluate patient satisfaction (CSQ-8)			X	X
Treatment Adherence Perception Questionnaire (TAPQ-adapt)			X	X
Positive and Negative Effects of Psychotherapy Scale (PANEPS-I)			X	X
Peritraumatic Dissociative Experiences Questionnaire (PDEQ-adapt)			X	X

### Sample size

In a meta-analysis published by Haug, Nordgreen, Öst, and Havik [20] on the effectiveness of iCBT interventions for treatment of anxiety, a mean effect size compared to waiting control groups of Hedges’ *g* = 0.55 (≈ *f* = 0.28) was found. A power analysis with the software G*Power [29] resulted in a target sample size of *N* = 103 to detect a medium-sized effect (*f* = 0.28) with *α* = 0.05 and a power of 0.80 for an ANCOVA. Using an assumed drop-out rate of 20% based on meta-analytic data on comparable studies [20], we will aim to recruit *N* = 128 participants.

### Recruitment

Information about the study will be given on the study’s website. Flyers and social media posts will be created. Interested study participants will be able to contact Sympatient Ltd. via the study website, by telephone, or by sending an e-mail. After verifying the technical requirements for participation via telephone (e.g., smart phone access), those who remain interested in participating in the study will be required to obtain a consultation report from their primary care physician prior to consenting to study participation to confirm that no physical exclusion criterion that could stand in the way of participation in the study (e.g., cardiovascular disease) is met. If the requirements are met, potential study participants will be referred to a clinician for a personal clinical diagnostic interview. This interview will be conducted in person or digitally via video call; participation in the study by telephone only is impossible. Inclusion is based on a classification diagnostic (DSM-5-SCID, American Psychiatric Association, 2014) that verifies the inclusion and exclusion criteria. After the inclusion and exclusion criteria have been verified, participants will receive a link to participate in the study on the digital study platform Qualtrics ®.

### Eligibility criteria

To be included, participants must meet all inclusion criteria and not fulfill any of the exclusion criteria listed below.

Inclusion criteria are as follows:

1. Age between 18 and 80
2. Provision of electronic informed consent
3. Possession of a smartphone with Internet access
4. Panic disorder as verified by DSM-5-SCID interview.
5. Panic symptoms experienced for at least 12 months

Exclusion criteria are as follows:

1. Acute suicidality as assessed by the DSM-5-SCID interview
2. Diagnosis of schizophrenia or a bipolar disorder as verified by the DSM-5-SCID interview

### Randomization

To ensure balance between treatment groups (intervention or CAU group), randomization will be performed using the randomizer in the Qualtrics® survey flow. The 128 planned study participants will be allocated to the two conditions with an automatic ratio adjustment via Qualtrics® according to a predetermined allocation ratio of 1:1. After the t1 assessment, participants will be automatically randomized and notified about their group allocation. Since the present study assesses outcome by self-report questionnaires, no blinded outcome assessors are needed. Data analysts will not be blinded to randomization. After random allocation to a study group, participants will stick with their group. Upon participant request, participants are allowed to leave at any time.

### Intervention

If a study participant is assigned to the intervention group, they will receive access to treatment with the Invirto right after baseline assessment (t1). Invirto provides iCBT for anxiety in eight modules with low-threshold therapeutic support. The contents of Invirto are in line with national treatment guidelines for anxiety disorders [6], which are based on established German and international CBT treatment manuals for anxiety disorders [30–33]. All content of Invirto is presented via an e-health tool (i.e., an app), including audio and video lessons recorded with experienced psychotherapists, behavioral exercises in virtual reality, and exercises for cognitive restructuring and transfer of content to everyday life. For their autonomous implementation of the app (e.g., at home), patients receive virtual reality glasses, headphones, instructions for use, and an access code for the Invirto app. The app contains approx. 15 h of content. In addition, all patients receive two video-conference individual sessions with clinical psychologists (each approx. 50 min). In the first personal contact, the therapist practices an initial guided exposure exercise with the patient and prepares them to continue self-directed exposures. Aside from the interactions within the app, there are no further prompting strategies or push notifications. Invirto uses regular check-ins; if the user appears suicidal, offers of help are provided. After all the content in the Invirto app has been completed, the treatment is reviewed, strategies for relapse prevention are discussed, and the user prepares with the therapist for the time after the treatment concludes in the second videoconference. Invirto ends with this final session, but patients still have access to the content of the Invirto app for one more year and are digitally prompted by the software to continue practicing.

### Measures

#### Primary outcome measures

##### Beck Anxiety Inventory (BAI)

The primary outcome is the improvement in anxiety symptoms as measured by the German version of the Beck Anxiety Inventory [34] from t0 to t1. The BAI is a self-report measure of anxiety severity that has 21 items on anxiety-associated sensations and cognitions, which are assessed in terms of their intensity over the previous week on a four-point Likert scale. The BAI scores are classified as minimal anxiety (0 to 7), mild anxiety (8 to 15), moderate anxiety (16 to 25), and severe anxiety (30 to 63). The BAI is regarded as reliable (*α* = 0.90) and valid as well as change sensitive [34, 35].

#### Secondary outcome measures

##### Panic and Agoraphobia Scale (PAS)

The Panic and Agoraphobia Scale (PAS; [36]) measures the symptom severity of panic disorder (with or without agoraphobia) within the past week. Thirteen items with a five-point Likert scale are used to assess five domains that limit quality of life in patients with panic disorder: panic attacks, agoraphobic avoidance, anticipatory anxiety, restriction, and health fears. Scores range from 0 to 52, with higher scores indicating higher levels of panic (0–8 = no panic, 9–18 = minimal panic, 19–28 = moderate panic, 29–39 = severe panic, and scores ≥ 40 = very severe panic). The PAS is considered a reliable (Cronbach’s *α* = 0.86) and valid test instrument [36]. In the present study, it will be assessed at t0, t1, and t2.

##### Beck Depression Inventory-II (BDI-II)

The Beck Depression Inventory-II (BDI-II; [37]) is a 21-item self-report questionnaire that assesses depressive symptoms over the previous 2 weeks. In the present study, it will be assessed at t0, t1, and t2. Scores range from 0 to 63, with higher scores indicating higher levels of depression (0–8 = no depression, 9–13 = minimal depression, 14–19 = mild depression, 20–28 = moderate depression, and 29–63 = severe depression). Internal consistency is good, with Cronbach’s *α* = 0.89 [38].

##### Questionnaire on panic-related Anxieties, Cognitions and Avoidance (ACA)

The Questionnaire on panic-related Anxieties, Cognitions and Avoidance (ACA [39]) is a self-report questionnaire consisting of three different subdomains: Body Sensations Questionnaire (BSQ; rating of the severity of 17 physical symptoms, such as palpitations), Agoraphobic Cognitions Questionnaire (ACQ; assessment of the frequency of 14 catastrophizing thoughts that occur when the person is anxious), and Mobility Inventory (MI; extent of avoidance of 27 situations that are often anxiety-provoking for patients with agoraphobia). The questionnaire shows good to very good consistencies and retest reliabilities (Cronbach’s *α* = 0.79–0.96). The instrument set has also been proved sensitive to the measurement of therapy effects [39]. In the present study, it will be assessed at t0, t1, and t2.

##### The Acceptance and Action Questionnaire-II (AAQ-II)

The Acceptance and Action Questionnaire-II (AAQ-II; [40]) is a questionnaire that aims to measure experiential avoidance and psychological flexibility, such as negative evaluations of feelings (e.g., “Anxiety is bad”) and avoidance of thoughts and feelings (e.g., “I try to suppress thoughts and feelings that I don’t like by just not thinking about them”). The questionnaire consists of seven items with a seven-point Likert scale from “(1) never true” to “(7) always true”. Internal consistency shows a Cronbach’s *α* of 0.84 for social phobia patients and 0.97 for students in the German version [41]. In the present study, it will be assessed at t0, t1, and t2.

##### Quality of Life—global item (WHOQOL-BREF)

The Quality of Life–global item (WHOQOL-BREF; [42]) is a cross-cultural questionnaire assessing generic quality of life. It was developed by the WHOQOL Group of the World Health Organization. The internal consistency of the questionnaire is acceptable (*α* = 0.7 [43]). For the present study, we will use the global QoL item, with answers ranging from “(1) very poor” to “(5) very good”, at t0, t1, and t2.

##### Client Satisfaction Questionnaire (CSQ-8)

The Client Satisfaction Questionnaire (CSQ-8, [44]) is a self-report questionnaire used to measure satisfaction with treatment at t1 and t2. The CSQ-8 consists of eight items formulated as questions, each with four given answer choices with no neutral position. The items are scored according to their ranking from “(1) most unfavorable” to “(4) most positive” and are summed up in a total score (scale range: 8–32). Good reliability and validity have been determined for the CSQ-8, in all studies or samples (Cronbach’s *α* ranging from 0.87 to 0.92; [44]).

##### Treatment Adherence Perception Questionnaire (TAPQ-adapt)

The Treatment Adherence Perception Questionnaire (TAPQ; [45]) is a self-report instrument for assessing patient perceptions and attitudes regarding their adherence to their medical treatment plans at t1 and t2. Originally developed for the somatic context, we have adapted the questionnaire by reformulating the items for the context of psychotherapy and the use of Internet-based interventions. The TAPQ has three scales: Perceived Behavior (six items, *α* = 0.89), Perceived Benefit (five items, Cronbach’s *α* = 0.85), and Perceived Burden (five items, Cronbach’s *α* = 0.84). The participants are asked about the recommended strategies in the therapy app. They are asked to select “yes” or “no” for each of the listed recommended strategies (e.g., expose oneself to fear-triggering situations) and are then asked about the perceived behavior, perceived benefit from it, and perceived burden of each strategy.

##### Positive and Negative Effects of Psychotherapy Scale for Internet-based Interventions (PANEPS-I)

The PANEPS-I [46], an adjusted version of the PANEPS [47] in which the wording of some items has been adapted, will be used in the current study at t1 and t2. The PANEPS-I is a self-report questionnaire assessing positive effects and adverse events during the individual’s most recent used digital intervention. The PANEPS-I is divided into four subscales with a total of 29 items: Positive Effects, Unethical Conduct, Malpractice, and Side Effects. All subscales of the PANEPS have strong internal consistency, with Cronbach’s *α* values ranging from 0.72 to 0.92 [46].

##### Adapted Peritraumatic Dissociative Experiences Questionnaire (PDEQ-adapt)

The PDEQ by Marmar et al. [48] is a standardized self-report 10-item questionnaire that retrospectively assesses dissociation, depersonalization, and derealization experiences during a traumatic event. Participants are asked to rate statements regarding dissociation on a five-point scale ranging from “(1) not at all true” to “(5) extremely true” at t1 and t2. An overall PDEQ score is calculated by summing the scores, with higher scores indicating more severe symptoms. Internal consistency shows a Cronbach’s *α* of 0.89 [48]. In the present study, we used an adapted version of the PDEQ (PDEQ-adapt) by reformulating the items to ask about dissociation during VR exposure instead of a past traumatic event.

### Data collection and management

Data collection and management will be carried out by the study center. All data for hypothesis testing will be collected online in clinically validated, standardized questionnaires (self-report) at three assessment points (t0 = baseline; t1 = three months after baseline; t2 = 6 months after baseline). For the online assessment, all participants will be encouraged to create an e-mail account that does not contain any personally identifying information. No additional personal information (such as names or addresses) will be gathered at t0, t1, or t2. Data security is guaranteed during all phases of the study. After confirming their participation with the digital consent form, the study participants will complete the study-related questionnaires to record the endpoints (BAI, PAS, WHOQOL-BREF, AAQ-II, BDI-II, ACA) for baseline assessment (t0). The survey instruments are presented in the “Measure” section. The baseline assessment takes approximately 20 to 30 min. Subsequently, participants will be randomly assigned to one of the two study groups (intervention group vs. CAU) and informed about this allocation. The participants in the intervention group will start the treatment with Invirto. The participants in the control group will receive care as usual (CAU). For post assessment (t1), the study participants will be asked to complete the questionnaires from t0 plus four additional ones (BAI, PAS, ACA, WHOQOL-BREF, AAQ-II, BDI-II, CSQ-8, PANEPS, TAPQ-adapt, PDEQ-adapt, IPQ, PANEPS-I). After that, the control group will be given access to the intervention Invirto. After a further 3 months, that is 6 months after baseline (t2), all participants will complete all the study questionnaires online once again (BAI, PAS, ACA, WHOQOL-BREF, AAQ-II, BDI-II, CSQ-8, PANEPS-I, TAPQ-adapt, PDEQ-adapt, IPQ, PANEPS-I). This is the end of the data collection in the study. For an overview of the assessment of all primary and secondary outcome measures, see Table 2. Trial methodology was planned according to the "Standard Protocol Items: Recommendations for Interventional Trials (SPIRIT)". For more information see Additional file 1. 

### Statistical analyses

The present study is designed to investigate the Invirto for patients with panic disorder. Intention-to-treat (ITT) analyses and complete case (CC) analyses are planned for statistical analyses. For the ITT analyses, this means that participants with all data available at t0 will be included in the statistical analysis and that missing values for t1 and t2 will be imputed using the multiple imputation (MI) procedure [49]. All hypothesis tests will be performed at a significance level of *α* = 0.05.

For primary analysis regarding symptom severity, data will be analyzed using analysis of covariance (ANCOVAs), with between-group differences over time (Invirto vs. CAU). Baseline scores will serve as covariates and group allocation as the independent variable. The dependent variable is the decrease in anxiety from time t0 to time t1 operationalized by the BAI sum score (primary outcome). The procedure for conducting the ANCOVA is guided by Field, Miles, and Field (2012). Before testing the hypothesis, the prerequisites of the ANCOVA will be checked.

The analysis regarding the secondary parameters will follow the analysis rationale of the primary outcome.

Partial eta square (η_p_^2^) and Cohen’s *d* will be calculated as effect sizes. Group differences at baseline will be assessed using chi-square tests and independent *t*-tests for continuous variables. All analyses will be conducted using IBM SPSS Statistics® 27.

### Ethical aspects and data safety

The clinical study has been approved by the local ethics committee (LPEK-0415). The present study’ concept as well as any subsequent amendments to the study concept are written in accordance with the Declaration of Helsinki as revised in October 2013 (by the 64th General Assembly in Fortaleza, Brazil). This clinical trial is being conducted in accordance with the published principles of Good Clinical Practice (ICH-GCP) guideline, EN ISO 14155:2011 + AC:2011. These principles include ethics committee procedures, patient education and informed consent, protocol adherence, administrative documentation, data collection, adverse event recording and reporting, and record retention. Prospective study subjects may only be enrolled in the clinical study after they have been informed in writing by the study center about the nature, significance, and scope of the clinical study in an appropriate and comprehensible manner and have given their voluntary consent. At the same time, the prospective study participants declare with their consent that they agree to the recording of data within the framework of the clinical study and to the data’s review. Study participants will be informed about the potential benefits and side effects of the intervention as well as the necessity and importance of a controlled clinical trial. Study participants may withdraw their consent at any time without giving a reason and without incurring any disadvantage. Their reasons for withdrawing from the study will be asked for and evaluated. If desired by the study participants, their collected data will be deleted. Otherwise, the data will be included in the evaluation. The consent will be kept in electronic form.

## Discussion

The present study aims to investigate the efficacy of the Internet-based intervention Invirto for panic disorder using a randomized controlled design. Evidence shows that Internet-based interventions can be effective in reducing anxiety symptoms [21]. Invirto aims at reaching those who do not seek conventional face-to-face therapy, thus narrowing the existing treatment gap. We expect that Invirto will be superior to CAU in reducing anxiety-related and depressive symptoms, overall quality of life, and avoidance behavior.

Still, the trial has some limitations that need to be acknowledged. First, the ratings regarding psychological symptomology (not diagnostic status) rely on self-ratings. While self-ratings come with several disadvantages (e.g., social desirability; [50]), they have the benefit of reducing drop-out rates due to fear of stigmatization, which may be particularly high in patients seeking help in digital interventions. Second, the study design’s inclusion of a CAU control group instead of an active control group comes with the disadvantage of not being able to eliminate the expectancy effect [51]. However, the common first step is to compare a treatment to a no-treatment waiting control group. Furthermore, the data analysts will not be blinded and there will be no external data management committee as this would require further funding for an independent data analysis. This might lead to the risk of bias in regard to data analysis.

Compared to traditional face-to-face therapy, Invirto has several benefits, including high flexibility of time and location and low-threshold access to digital psychotherapy without long waiting periods. In addition, Invirto has the major strength of being one of the first programs to include self-managed exposure therapy in VR. Therefore, the role of self-applied VR for exposure therapy in digital psychotherapy will be highlighted in the study. Furthermore, Invirto is based on CBT, which is the guideline-recommended psychotherapy for panic disorder [7].

Future practical implications and potential possibilities of Internet-based interventions for anxiety disorders will be discussed. Invirto may be able to help those who cannot access psychotherapy (due, for example, to geographical distance or long waiting times) or who avoid face-to-face-therapy due to stigma.

### Timetable and research plan

The proposed study began in 2022 and will be completed in 2023. The recruitment has already started, and 115 participants have already been randomized. The recruitment and treatment will continue through summer 2023. The study protocol was created in June 2022. During the second half of 2023, the follow-up assessment will be carried out. Thereafter, data will be prepared and analyzed.

### Trial status

The first participant was enrolled in February 2022. Currently, 115 participants have completed their baseline assessment. At the time of submission of this study protocol, participants were still being recruited and no data had yet been extracted or analyzed. All future changes to the study protocol will be recorded in an independent amendment. SPIRIT guidelines were followed for the whole article.

### Supplementary Information

**Additional file 1: Table S1.** SPIRIT 2013 Checklist.

## Data Availability

Data not available yet but will be made available upon request once results are published. The German version of model of consent form can be shared upon request. No biological specimens were collected.

## Abbreviations

AAQ-II: The Acceptance and Action Questionnaire-II
ACA: Questionnaire on panic-related Anxieties, Cognitions and Avoidance
ANCOVA: Analysis of covariance
BAI: Beck Anxiety Inventory
BDI-II: Beck Depression Inventory-II
CAU: Care as usual
CBT: Cognitive behavioral therapy
CC: Complete case
CSQ-8: Questionnaire to Evaluate Patient Satisfaction
iCBT: Internet-based cognitive behavioral therapy
ITT: Intent-to-treat
PANEPS-I: Positive and Negative Effects of Psychotherapy Scale
PAS: Panic and Agoraphobia Scale
PDEQ-adapt: Peritraumatic Dissociative Experiences Questionnaire (adapted version)
TAPQ-adapt: Treatment Adherence Perception Questionnaire (adapted version)
VR: Virtual reality
WHOQOL-BREF: Quality of Life–global item
